# Needle angle dynamics as a rapid indicator of drought stress in *Larix kaempferi* (Lamb.) Carrière: advancing non-destructive imaging techniques for resilient seedling production

**DOI:** 10.3389/fpls.2025.1550748

**Published:** 2025-05-12

**Authors:** Ukhan Jeong, Dohee Kim, Sohyun Kim, Seung Hyun Han, Eun Ju Cheong

**Affiliations:** ^1^ Plant Genetics and Breeding Lab, Department of Forest and Environment System, College of Forest and Environmental Science, Kangwon National University, Chuncheon, Republic of Korea; ^2^ Forest Technology and Management Research Center, National Institute of Forest Science, Pocheon, Republic of Korea

**Keywords:** *Larix kaempferi*, forest nursery, drought stress monitoring, early detection, leaf angle, phenomics

## Abstract

*Larix kaempferi* (Lamb.) Carrière, a valuable species for timber production and reforestation, faces challenges in large-scale seedling propagation due to its slow growth cycle and high susceptibility to environmental stressors. Early detection of drought stress is critical for preparing seedlings for harsh field conditions and for optimizing irrigation strategies. This study aimed to detect drought stress at an early stage in *L. kaempferi* seedlings by integrating physiological traits with image-based phenotypic measurements, with a focus on needle angle dynamics under controlled drought and irrigation conditions. The apical needle angle of one-year-old seedlings was measured using ImageJ, while seedling-level analysis was conducted using PlantCV to collect data and extract relevant parameters. Statistical analyses were performed to evaluate temporal trends and to identify growth environment and physiological traits significantly influenced by drought stress. As a result, apical needle wilting and recovery, along with seedling-level image analysis (parameter: Center of Mass(y)), exhibited significant responses to drought stress as early as Day 2. This provides a non-destructive method for early detection, preceding observable changes in physiological traits such as chlorophyll fluorescence and needle temperature that responded to drought stress by Day 6, as well as before seedling mortality occurred. Multiple regression analysis indicated that, as drought stress progressed, solar radiation and thermal-related parameters (ФNPQ and needle temperature) emerged as key predictors of needle angle variation. Image-based approaches, including RGB and thermal imaging, proved effective for real-time stress monitoring, demonstrating their practical potential for nursery applications. In summary, this study lays the groundwork for needle-based phenomic approaches using imaging techniques in nursery systems and highlights the need for further research to optimize these methods for the large-scale, cost-effective production of high-quality, drought-resilient *L. kaempferi* seedlings.

## 1 Introduction

*Larix kaempferi* (Lamb.) Carrière (Japanese larch) is a deciduous conifer belonging to the *Pinaceae* family, predominantly distributed in East Asia (Nagaike et al., 2003; Choi et al., 2021; Wu et al., 2021). In Korea, *L. kaempferi* is widely valued for its use as timber and as a key species for reforestation (Kim et al., 2013). However, despite its high demand, the extended larch seed production cycle of at least 2 to 3 years makes it difficult to meet demand without man-made processes (Kaliniewicz et al., 2012). Large-scale seedling production using controlled nursery systems is a viable solution to this challenge. Nonetheless, such systems remain labor-intensive and costly, necessitating improvements in production structure to ensure broader adoption.

Global warming and climate change intensify environmental stresses, significantly impacting *L. kaempferi* growth, physiology, and ecology (Wu et al., 2021). Among these, water is vital for all organisms, with prolonged drought stress having particularly adverse effects on plants (Trenberth et al., 2014; Sarris and Christodoulakis, 2024). In particular, seedlings, being young and vulnerable, are especially prone to drought-induced damage (Khurana and Singh, 2004; Galvez et al., 2011; Fernández et al., 2014). When transplanted from controlled nursery environments to field conditions, seedlings are exposed to severe stresses, necessitating adequate preparation (Allen et al., 2017; South et al., 2023). Priming, a method of inducing controlled stress, has been reported to enhance drought tolerance. This process involves exposing plants to mild stress conditions, such as limited water availability, to trigger adaptive responses that improve resilience to subsequent, more severe stress (Grossnickle, 2012; Abid et al., 2016; Abdallah et al., 2017; Tankari et al., 2021; Sintaha et al., 2022; Ru et al., 2022). Therefore, the development of techniques to diagnose drought stress at early stages is crucial for preparing seedlings for climate-related challenges and for implementing efficient irrigation systems alongside priming treatments.

Phenotypes refer to the observable traits of organisms, such as their physical structure, physiological processes, and responses to environmental factors. By defining and quantifying tree stress through phenotypic measurements, subjective evaluation criteria can be standardized and objectified. This data can be used to promptly assess plant health and respond effectively. The imaging-based plant phenomics, which comprehensively analyzes phenotypic traits along with genetic and environmental characteristics, has been gaining attention for its applicability across various fields (Kumar et al., 2015; Tardieu et al., 2017; Perez-Sanz et al., 2017; Pasala and Pandey, 2020). Depending on the method, high-cost equipment (e.g., 3D imaging, hyperspectral imaging) allows for precise measurements but requires technical expertise (Forsström et al., 2021; Pisek et al., 2023). On the other hand, while low-cost equipment (e.g., RGB imaging, thermal imaging) may have limitations in precision, they can still be supplemented based on their intended use and are more practical for widespread adoption (Cozzolino, 2023; Naqvi et al., 2024). In plant stress research, RGB imaging is widely used with unmanned aerial vehicles (UAVs) and thermal cameras to measure drought stress (Zhang et al., 2019a; Su et al., 2020; De Swaef et al., 2021; Chandel et al., 2022). However, early-stage stress detection is challenging using RGB-based vegetation indices, making them more suitable for field monitoring than for precise, real-time measurements at the individual plant level. Additionally, long-term monitoring studies integrating morphological parameters often face limitations in real-time stress detection (Zhang et al., 2019b; Bhusal et al., 2020, 2021; Yasin et al., 2024).

Leaf movement is a key physiological mechanism that supports plant survival, offering insights into a plant’s adaptability to its environment and tolerance to abiotic stress (Yang et al., 2023). Accordingly, leaf angle measurements offer a promising alternative for early drought stress detection (Ehleringer and Comstock, 1987; Yavas et al., 2024). In controlled nursery environments, where external factors such as wind are minimized, the utility of leaf angle measurements is expected to increase. Advancements in real-time leaf angle monitoring techniques further enhance its applicability (Kenchanmane Raju et al., 2020; Geldhof et al., 2021; Kattenborn et al., 2022; Jiang et al., 2024; Oskam et al., 2024). Recently, leaf angle has been targeted in breeding programs aimed at improving crop yield, leading to an increasing number of studies integrating leaf angle traits with agronomic research (Ji et al., 2024; Liu et al., 2024; Wu et al., 2024). However, research on the correlation between leaf angle and tree physiological traits (e.g., chlorophyll content, chlorophyll fluorescence, photosynthesis, and water status) remains limited, emphasizing the need for further investigation, particularly from the perspective of tree breeding. Moreover, tracking needle angle in conifers presents a significant challenge, highlighting the necessity for research aimed at developing more accessible and efficient measurement methods.

This study aimed to identify suitable parameters for early diagnosis of drought stress in *L. kaempferi* seedlings by exploring image-based changes in needle angles. Needle angle is particularly useful because it responds rapidly to changes in water availability, reflecting early physiological stress before visible symptoms such as wilting occur. This makes it an effective and non-destructive indicator for detecting drought stress at early stages. To quantify drought stress objectively, the study analyzed apical needle angles and individual seedling images, alongside greenhouse environmental parameters and tree physiological parameters, such as chlorophyll content, chlorophyll fluorescence, electrical conductivity, and needle temperature parameters.

## 2 Materials and methods

### 2.1 Plant materials

The study used 1-year-old *L. kaempferi* seedlings (mean height: 24.3 ± 3.3 cm, mean root collar diameter: 2.8 ± 0.4 mm), produced at the Forest Technology and Management Research Center, National Institute of Forest Science (37°45’39”N, 127°10’13”E). Seeds were submerged for 2 days in mid-April 2024 and sown at a rate of three seeds per 320 mL container filled with a growth medium (peat moss: perlite: vermiculite = 1:1:1, v/v). From April to August, the seedlings were irrigated daily with 20 L/m² using a sprinkler system. From June onward, seedlings were fertilized weekly with a 1 g/L (1,000 ppm) solution of MultiFeed 19 (19N:19P_2_O_5_:19K_2_O; Haifa Chemicals, Haifa, Israel) alongside irrigation. On August 8, seedlings underwent acclimatization in a greenhouse at the College of Forest and Environmental Sciences, Kangwon National University (37°52’00”N, 127°44’51”E).

### 2.2 Experimental conditions

The drought stress experiment was conducted over a 7-day (BD: before drought to D6: Day 6 of the experiment) period from August 16 to August 22, 2024, in a greenhouse at Kangwon National University. A total of 60 seedlings were randomly selected, with 10 seedlings per treatment chosen for soil temperature (ST) and moisture (SM) with electrical conductivity (EC) (n = 10), physiological (n =10), and image-based measurements (n =10). Treatments were as follows:

· Control (n = 30): irrigated daily with 2.35 L/container between 11:00 AM and 12:00 PM.
· Drought (n = 30): irrigated the same as the control and no irrigation from D1.

Greenhouse air temperature (AT), humidity (AH), and solar radiation (SR) were continuously monitored using Juns OL sensors (PurumBio, Suwon, Korea). Soil temperature and moisture were recorded at 30 min intervals using a Hobo micro station (H21-USB, Onset, Bourne, MA, USA) equipped with temperature (S-SMD-M005) and moisture sensors (S-TMB-M002).

### 2.3 Physiological measurements

Chlorophyll index (SPAD) and fluorescence (Fm’: maximum fluorescence in light-adapted state, Fo’: minimum fluorescence in light-adapted state, Fv’/Fm’: maximum quantum yield of PSII in light-adapted state, ΦII: quantum yield of PSII, ΦNO: quantum yield of non-regulated energy dissipation, ΦNPQ: quantum yield of regulated energy dissipation, PSIact: PSI activity, PSIopen: open reaction centers in PSI, qL: fraction of open PSII reaction centers) were measured using a MultispeQ V2.0 (PhotosynQ, East Lansing, MI, USA). Measurements were repeated five times per seedling across different needle bundles every two days, starting from D2 of the experiment (between 12:00 PM and 6:00 PM). EC was monitored in real time using Juns OL sensors (PurumBio, Suwon, Korea), with electrodes attached to the seedling stem, 3 cm above the soil surface.

### 2.4 Image-based measurements

To collect RGB images for needle angle measurements, side-view images of control (n = 2) and drought-treated seedlings (n = 2) were captured together in a single frame (n = 4). Five images were simultaneously taken at 10 min intervals using a time lapse camera (ATL200S, Afidus, New Taipei City, Taiwan). The camera was positioned at the same height as the target pots, 60 cm away from the seedlings, with a black background placed behind the seedlings to minimize visual interference. To reduce image distortion, wide-angle, digital zoom, image stabilization, and automatic exposure optimization functions were turned off, and images were captured at a 1:1 aspect ratio. Thermal images were obtained using a thermal camera (PI 160i, Optris, Berlin, Germany) at approximately 2–3 m distance, starting from 12:00 PM, aligning with the RGB image collection.

### 2.5 Data processing

#### 2.5.1 Needle angle measurements

**Figure 1:**
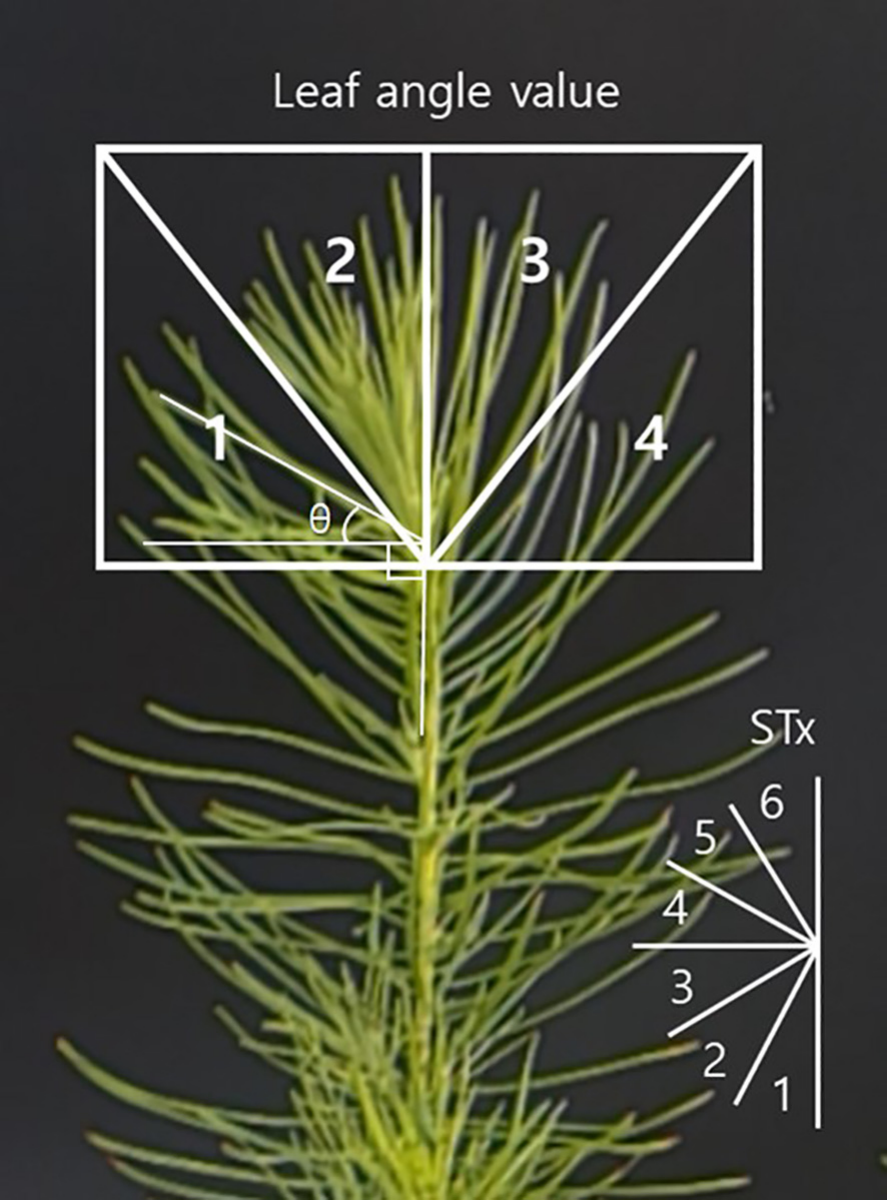
Criteria for collecting needle angle data.

In this study, needle angles at the apical part of seedling, which respond rapidly to drought stress, were primarily measured. The needle angle (θ) was defined as the interior angle (± 90°) formed by drawing a horizontal line from the base of the needle to its tip (**Figure 1**). However, since it is challenging to track a single needle consistently over time, and individual measurements may vary significantly, the apical images were divided into four quadrants. Needle angles within each quadrant were measured using ImageJ software ver. 1.53k (NIH, Bethesda, MD, USA), and the average value was taken as the resultant needle angle.

The needle angles exhibited significant changes at 9:00 AM and 6:00 PM, which led to data being collected twice per day (CM: control morning, CE: control evening, DM: drought treatment morning, DE: drought treatment evening). Seedlings that became completely discolored and wilted during the experiment were excluded from the daily data analysis (included only if the apical part is measurable). The analysis continued until at least 30 needle angle values were available from surviving seedlings in the drought treatment for RMANOVA analysis. Needle angle parameters were categorized as follows:

· STx: needle angle ranges divided into six intervals (ST1: – 90° ≤ θ < – 60°, ST2: – 60° ≤ θ < – 30°, ST3: – 30° ≤ θ < 0°, ST4: 0° ≤ θ < + 30°, ST5: + 30° ≤ θ < + 60°, ST6: + 60° ≤ θ ≤ + 90°).
· BD-M: needle angle before drought treatment – current needle angle.
· PM-M: previous needle angle – current needle angle (e.g., Day 1 evening – Day 2 morning, Day 2 morning – Day 2 evening).
· PM-M(ST): previous needle angle measured at the same time of day – current needle angle (e.g., Day 1 morning – Day 2 morning, Day 1 evening – Day 2 evening).

#### 2.5.2 Seedling level measurements

**Figure 2:**
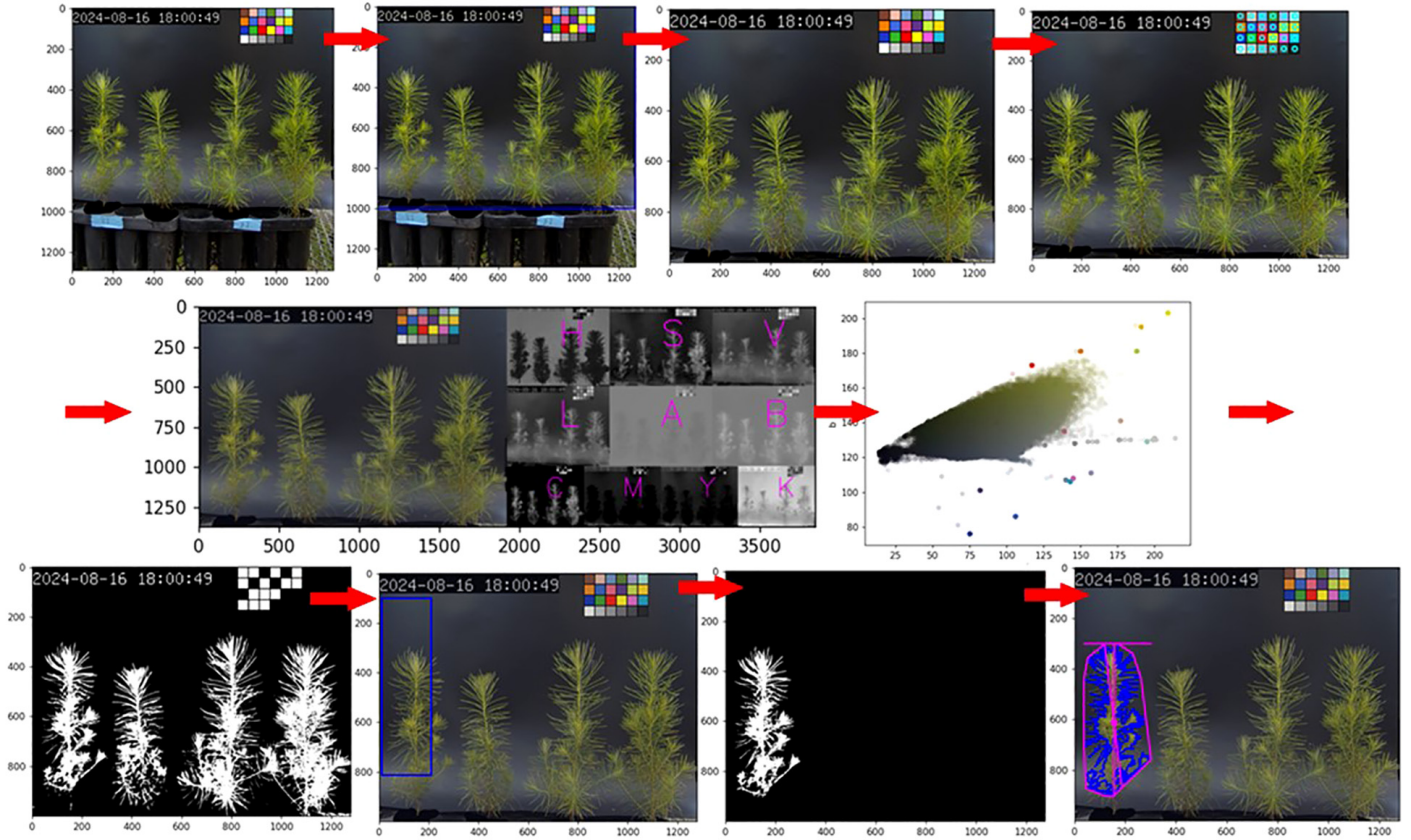
Seedling level parameter extraction process using PlantCV (color correction, mask creation, and data extraction). The extracted parameters were analyzed using the Kruskal-Wallis test across different days and treatments, followed by the Mann-Whitney test with Bonferroni correction for *post-hoc* analysis (P < 0.05).

**Figure 3:**
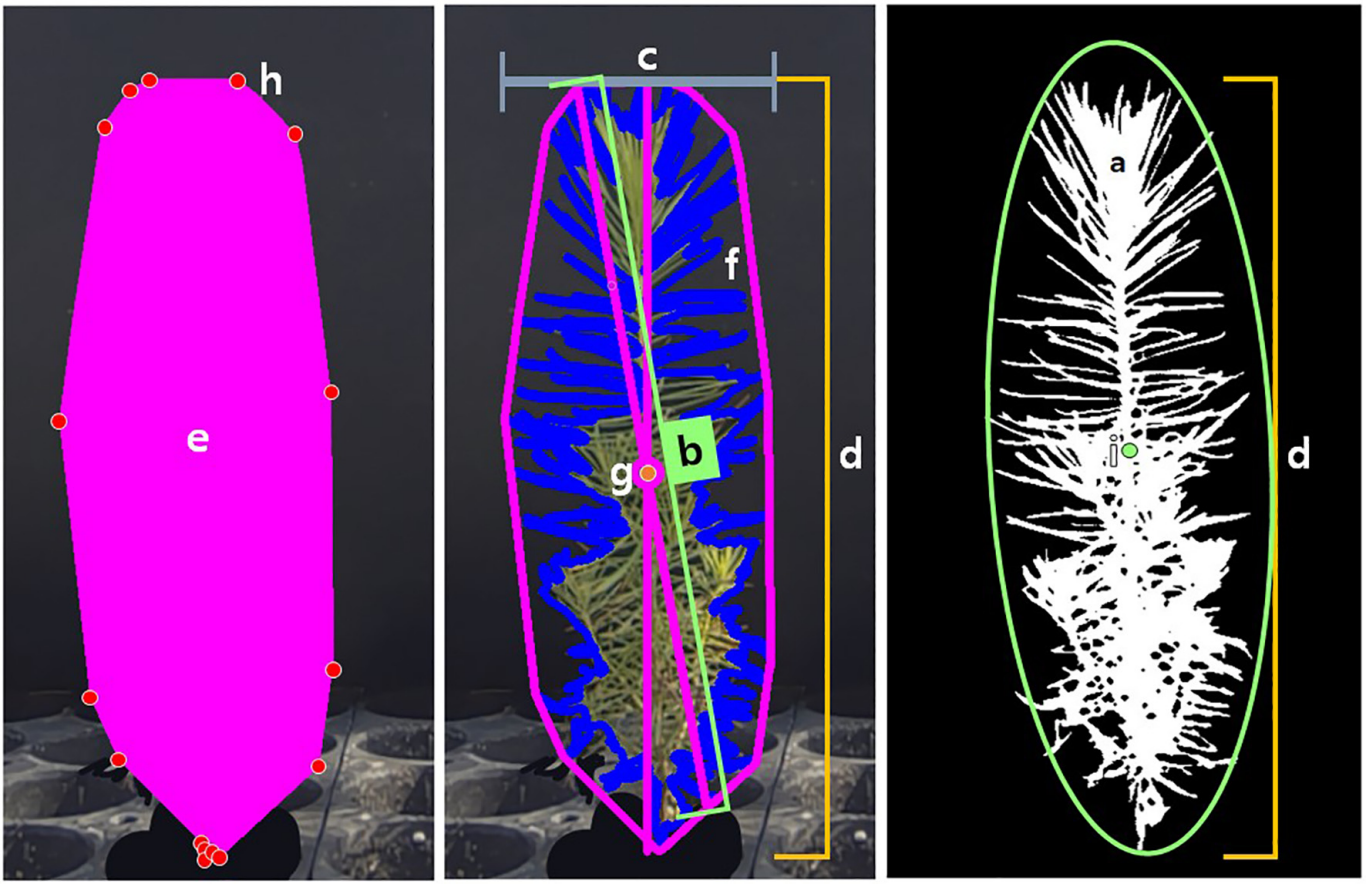
Criteria for seedling level parameters. **(a)** Area (surface area); **(b)** Longest Path (longest distance passing through the center); **(c)** Width (maximum horizontal length); **(d)** Height (maximum vertical length); **(e)** Convex Hull Area (area of the polygon connecting the outermost points); a/e: Solidity (density, calculated as the ratio of Area to Convex Hull Area); **(f)** Perimeter (outline of the mask); **(g)** Center of Mass(x,y) (center point of all pixels); **(h)** Convex Hull Vertice (number of vertices in the polygon connecting the outermost points); **(i)** Ellipse Center(x,y) (center of the ellipse fitted to the outline).

An image analysis method using the Python-based PlantCV framework (https://plantcv.readthedocs.io/en/stable/) was applied (Gehan et al., 2017). The analysis protocol is as follows (**Figure 2**):

· Color correction: the range of images to be used was set, and the image colors were corrected based on a color chart. By standardizing the colors to reduce the influence of lighting, this process helped mitigate distortions of the plant in the image, enabling a more accurate analysis.
· Mask creation: a mask was created to distinguish the plant from the background, defining the range for the computer to analyze. After color correction, a suitable color range for the plant was set based on color distribution, and a new image was created that matched the plant’s shape in the original image. In the mask image, pixels within the defined color range were converted to white (255, 255, 255), while pixels outside the range were converted to black (0, 0, 0), resulting in a binary image. Noise was then removed using the fill and fill_holes functions. In this study, as each image contained four plants, an additional step was taken to create an overall mask image and then separate it into individual plant masks. The desired range (object) was defined to generate masks for each plant. This range included not only the defined rectangular area but also any connected objects outside the rectangle within the range.
· Data extraction: the prepared image and mask were compared to extract image information for the selected plants. The extracted data was based on the pixels and coordinates within the image. The range measured could be confirmed through the returned images after extraction. As traditional data extraction methods were often difficult for users to interpret, only the necessary data were selected and extracted. In this study, the extracted parameters included Area (surface area), Longest Path (longest distance passing through the center), Width (maximum horizontal length), Height (maximum vertical length), Convex Hull Area (area of the polygon connecting the outermost points), Solidity (density, calculated as the ratio of Area to Convex Hull Area), Perimeter (outline of the mask), Center of Mass(x, y) (center point of all pixels), Convex Hull Vertice (number of vertices in the polygon connecting the outermost points), and Ellipse Center(x, y) (center of the ellipse fitted to the outline) (**Figure 3**).

#### 2.5.3 Needle temperature parameters

Needle temperature was measured using IR camera software PIX Connect Rel. 3.6.3046.0 (Optris, Berlin, Germany) by labeling clustered needle areas and collecting the average needle temperature within those areas. Needle (leaf) temperature parameters, including vapor pressure deficit (VPD) (Stull, 2015; Grossiord et al., 2020), crop water stress index (CWSI), and leaf temperature difference (LTD), were derived from the collected needle temperature data (Gardner et al., 1992; Zhou et al., 2021). Needle temperature parameters are calculated using the following Equations 1-6:


(1) VPD=LVP−AVP

(2) $$\text{LVP}=0.61078\times \text{exp}(\frac{17.27\times Tl}{Tl+237.3})$$

(3) $$\text{AVP}=0.61078\times \text{exp}(\frac{17.27\times Ta}{Ta+237.3})\times (\frac{AH}{100})$$

(4) $$\text{CWSI}(\text{Tl})=\frac{Tl-Tlw}{Td-Tlw}$$

(5) $$\text{CWSI}(\text{Tl}-\text{Ta})=\frac{\left(Tl-Ta)-(Tlw-Taw\right)}{\left(Tld-Tad)-(Tlw-Taw\right)}$$

(6) LTD=Average Tl of control−Tl of drought treatment

The meanings of each abbreviation are as follows; LVP: leaf vapor pressure, AVP: air vapor pressure, Tl: leaf temperature, Ta: air temperature, AH (RH): air humidity (relative humidity), Tlw: minimum leaf temperature, Tld: maximum leaf temperature, Taw: minimum air temperature, Tad: maximum air temperature.

### 2.6 Statistical analysis

A 2-way RMANOVA (repeated measures analysis of variance) was conducted for physiological traits (n = 30), whereas a 3-way RMANOVA was performed for needle angle parameters (n = 30). *Post-hoc* analysis was carried out using pairwise t-tests with Bonferroni correction (P < 0.05). For seedling level parameters (n ≤ 10), the Mann-Whitney U test with Bonferroni correction (P < 0.05) was used as a *post-hoc* analysis following the Kruskal-Wallis test. Stepwise multiple regression analysis was performed using needle angle parameters as dependent variables and the remaining parameters as independent variables. The significant predictors identified through the stepwise multiple regression analysis were further analyzed with principal component analysis (PCA) alongside the needle angle parameters. Statistical analyses, including RMANOVA, t-test, non-parametric tests, and stepwise multiple regression, were conducted using SPSS ver. 26 (IBM, Armonk, NY, USA), while PCA was performed using R ver. 3.6.1 (R Core Team, Vienna, Austria) with the ‘psych’ package for analysis and the ‘ggbiplot’ package for visualization.

## 3 Results

### 3.1 Growing conditions

**Figure 4:**
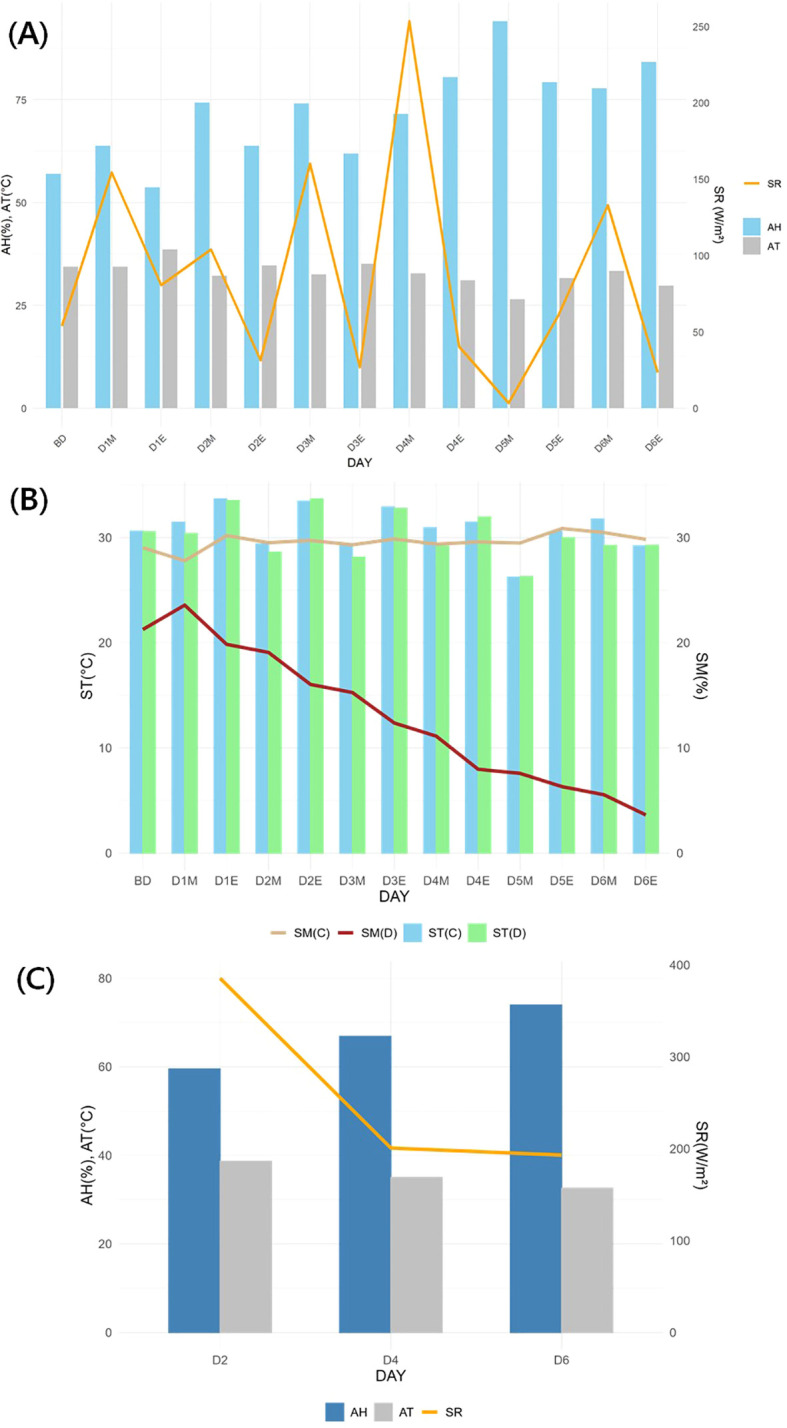
Greenhouse and soil environment during the experiment. **(A)**: Temporal atmospheric environment; **(B)**: Temporal soil environment; **(C)**: Atmospheric environment (9:00 AM – 6:00 PM) during physiological traits measurement. AH, air humidity; AT, air temperature; SR, solar radiation; ST, soil temperature; SM, soil moisture; BD, before drought treatment; DOM, Day O of the experiment at 9:00 AM; DOE, Day O of the experiment at 6:00 PM; C, Control, D, Drought treatment.

During the experiment, the greenhouse conditions showed an average AH of 79.13 ± 13.88%, AT of 30.46 ± 4.72°C, and SR of 194.32 ± 233.92 W/m² (**Figure 4**). SM in the control remained stable at 29.48 ± 1.22%, whereas the drought treatment decreased to 13.97 ± 6.75%, showing a declining trend. ST showed no significant differences between the treatments, with 29.55 ± 4.06°C in the control and 29.45 ± 3.63°C in the drought treatment. Excluding rainfall on D5, SR and AH were higher in the morning compared to the evening, while AT exhibited the opposite trend.

### 3.2 Physiological traits

Chlorophyll fluorescence parameters that exhibited an interaction effect between day and treatment included Fm’, Fv’/Fm’, ΦII, ΦNO, and ΦNPQ (P < 0.01) (**Table 1**, **Supplementary Table S1**). No parameters exhibited a main effect of treatment alone, whereas Fv’/Fm’, ΦII, and ΦNPQ showed a main effect of day only. On D2, SR reached its highest level during the experimental period, suggesting that the control was exposed to slightly higher light and heat stress than the drought treatment (**Figure 4C**). On D2, significant differences between the two treatments were observed only in Fm’ (P < 0.01) and Fo’ (P < 0.001), and the control showed signs of recovery over time (**Table 1**, **Supplementary Table S2**). In contrast, on D6 in the drought treatment, a significant decrease in Fv’/Fm’ and a significant increase in ΦNPQ were observed over time, indicating drought stress. Meanwhile, SPAD showed no significant differences (P > 0.05). On the other hand, EC showed significant differences (P < 0.001) between the two treatments starting from day 2 due to root pressure and osmotic effects from direct irrigation. Needle temperature parameters tended to be more sensitive than chlorophyll fluorescence. Except for CWSI(Tl-Ta), an interaction effect between day and treatment, as well as the main effects of day and treatment, was identified. From D4, all needle temperature parameters showed significant differences (P < 0.01) between the two treatments. A clear drought stress over time was observed in LTD on D4, while VPD and CWSI(Tl) exhibited a pattern similar to chlorophyll fluorescence, appearing on the D6.

**Table 1:** Statistical results of physiological traits.

	Day 2		Day 4		Day 6	
	Control	Drought	Control	Drought	Control	Drought
Fm' (D×T)	3882.83 ± 1294.82B	2984.77 ± 771.87	3912.65 ± 1119.77B	2932.72 ± 906.06	5033.28 ± 1247.16A	2722.64 ± 909.69
	**		***		***	
Fo'	1120.37 ± 319.85AB	765.97 ± 191.83	988.07 ± 262.59B	741.37 ± 189.79	1273.10 ± 293.79A	839.07 ± 196.80
	***		***		***	
Fv'/Fm' (D×T)	0.70 ± 0.06B	0.74 ± 0.03a	0.74 ± 0.02A	0.74 ± 0.02a	0.75 ± 0.01A	0.66 ± 0.14b
	–					
ΦII (D×T)	0.64 ± 0.06B	0.69 ± 0.03a	0.69 ± 0.02A	0.69 ± 0.03a	0.69 ± 0.02A	0.60 ± 0.14b
	–					
ΦNO (D×T)	0.18± 0.03	0.18 ± 0.02a	0.18 ± 0.02	0.18 ± 0.02a	0.19 ± 0.01	0.16 ± 0.04b
	ns		ns		**	
ΦNPQ (D×T)	0.19 ± 0.08A	0.13 ± 0.04b	0.12 ± 0.03B	0.13± 0.04b	0.12 ± 0.02B	0.24 ± 0.17a
	–					
PSIact	1.36 ± 1.08	2.48 ± 2.31	2.92 ± 7.53	1.87 ± 1.85	1.92 ± 1.10	0.95 ± 1.01
	–					
PSIopen	0.46 ± 0.71	0.85 ± 1.16	0.81 ± 0.92	0.52 ± 0.37	0.61 ± 0.68	0.74 ± 0.53
	–					
qL	0.76 ± 0.08	0.79 ± 0.06	0.77 ± 0.07	0.78 ± 0.08	0.76 ± 0.06	0.79 ± 0.09
	–					
SPAD	18.18 ± 9.75	15.09 ± 10.17	18.89 ± 8.70	19.30 ± 9.35	17.33 ± 8.68	14.04 ± 10.15
	–					
EC (D×T)	0.13 ± 0.00B	0.11 ± 0.00a	0.12 ± 0.00C	0.09 ± 0.00c	0.14 ± 0.00A	0.10 ± 0.00b
	***		***		***	
VPD (D×T)	3.59 ± 1.01A	3.55 ± 0.97b	3.49 ± 0.64A	4.44 ± 0.76a	0.86 ± 0.30B	1.35 ± 0.31c
	ns		***		***	
CWSI(Tl) (D×T)	0.50 ± 0.30A	0.48 ± 0.29b	0.25 ± 0.19B	0.54 ± 0.21b	0.32 ± 0.19B	0.72 ± 0.16a
	ns		***		***	
CWSI(Tl-Ta) (D×T)	0.45 ± 0.38	0.46 ± 0.29	0.31 ± 0.23	0.53 ± 0.25	0.23 ± 0.23	0.50 ± 0.30
	ns		**		***	
LTD (D×T)	0.18± 2.24	0.36 ± 2.20a	0.42 ± 1.56	-1.94 ± 1.71b	0.08 ± 0.80	-1.57 ± 0.66b
	ns		***		***	

Mean ± SD (n = 30). 2-way RMANOVA was conducted, followed by pairwise t-tests with Bonferroni correction for *post-hoc* analysis (P < 0.05). When the day × treatment interaction effect is significant, the parameter is marked with “(D×T)” (P < 0.05), and *post-hoc* analysis results were provided in **Supplementary Tables 1**, **2**. When the day main effect is significant, comparisons are made separately for control (uppercase letters) and drought (lowercase letters) (P < 0.05). When the treatment main effect is significant, comparisons are conducted for Day 2, Day 4, and Day 6, with significance levels denoted as follows: “ns” (P > 0.05), “**” (P < 0.01), “***” (P < 0.001). Fm', maximum fluorescence in light-adapted state; Fo', minimum fluorescence in light-adapted state; Fv'/Fm', maximum quantum yield of PSII in light-adapted state; ΦII, quantum yield of PSII; ΦNO, quantum yield of non-regulated energy dissipation; ΦNPQ, quantum yield of regulated energy dissipation; PSIact, PSI activity; PSIopen, open reaction centers in PSI; qL, fraction of open PSII reaction centers; SPAD, Chlorophyll index; EC, electrical conductivity; VPD, vapor pressure deficit; CWSI, crop water stress index; Tl, leaf temperature; Tl-Ta, leaf temperature – air temperature; LTD, leaf temperature difference.

### 3.3 Needle angle

#### 3.3.1 Apical vigor

**Figure 5:**
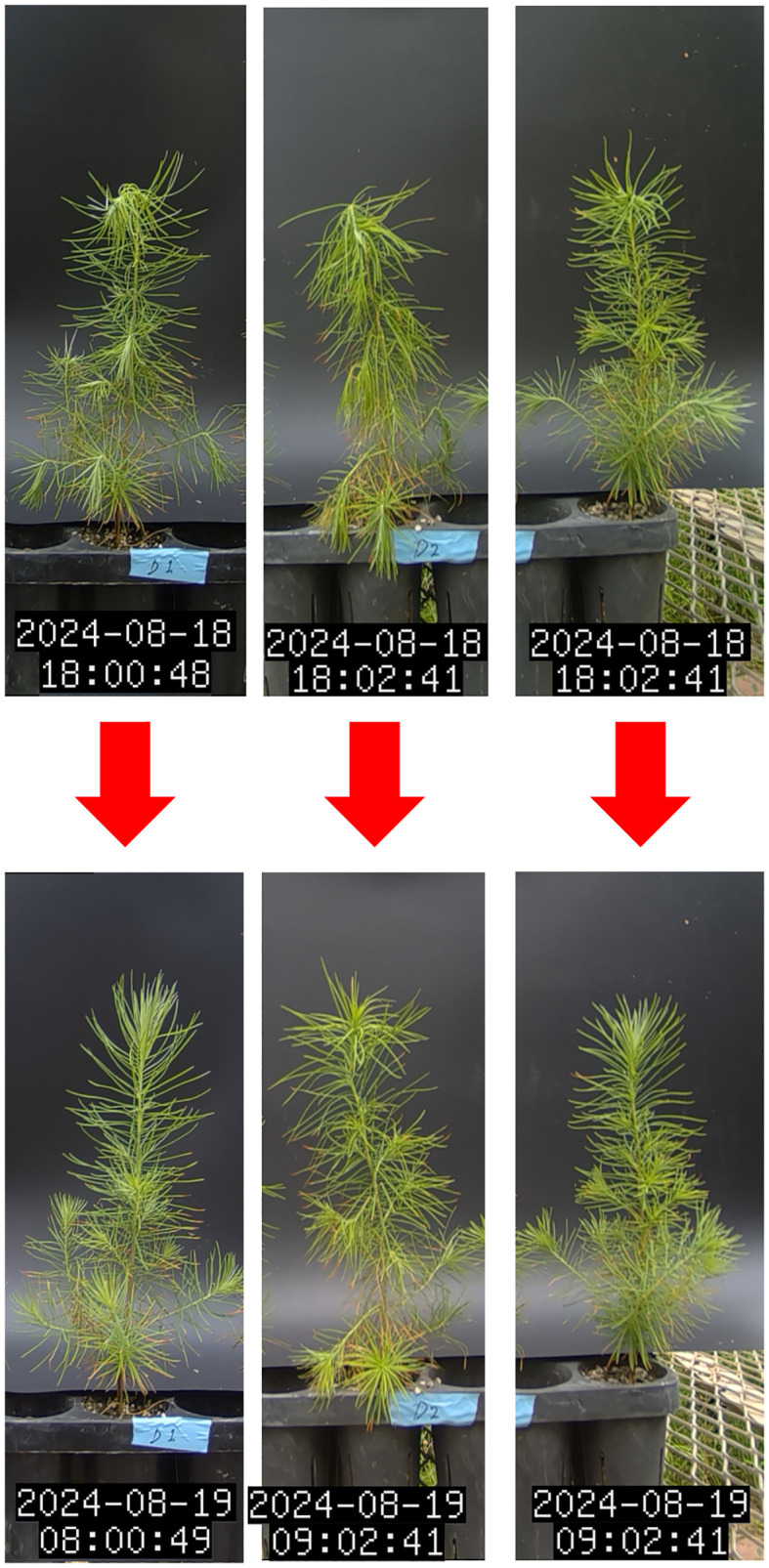
Time-lapse of *L. kaempferi* under drought stress conditions (D2E to D3M).

The first signs of wilting were detected on D2E (evening of D2), occurring earlier than changes in chlorophyll fluorescence and needle temperature parameters (**Table 2**, **Figure 5**). During prolonged drought stress, 70% of the apical parts of the seedlings wilted in the evening but recovered by the following morning. However, these seedlings completely died within 1–2 days. In contrast, the remaining 30% of seedlings did not recover at the apical part and died without any signs of recovery.

**Table 2:** Vitality of the apical part of the seedling.

	D2E	D3M	D3E	D4M	D4E	D5M	D5E	D6M	D6E
○	60	90	50	60	30	50	50	50	10
△	30	0	10	0	20	0	0	0	10
X	10	10	40	40	50	50	50	50	80

D: day; M: morning; E: evening; ○: survival; △: recovery the next day after the first wilting; X: completely died.

#### 3.3.2 RMANOVA

**Figure 6:**
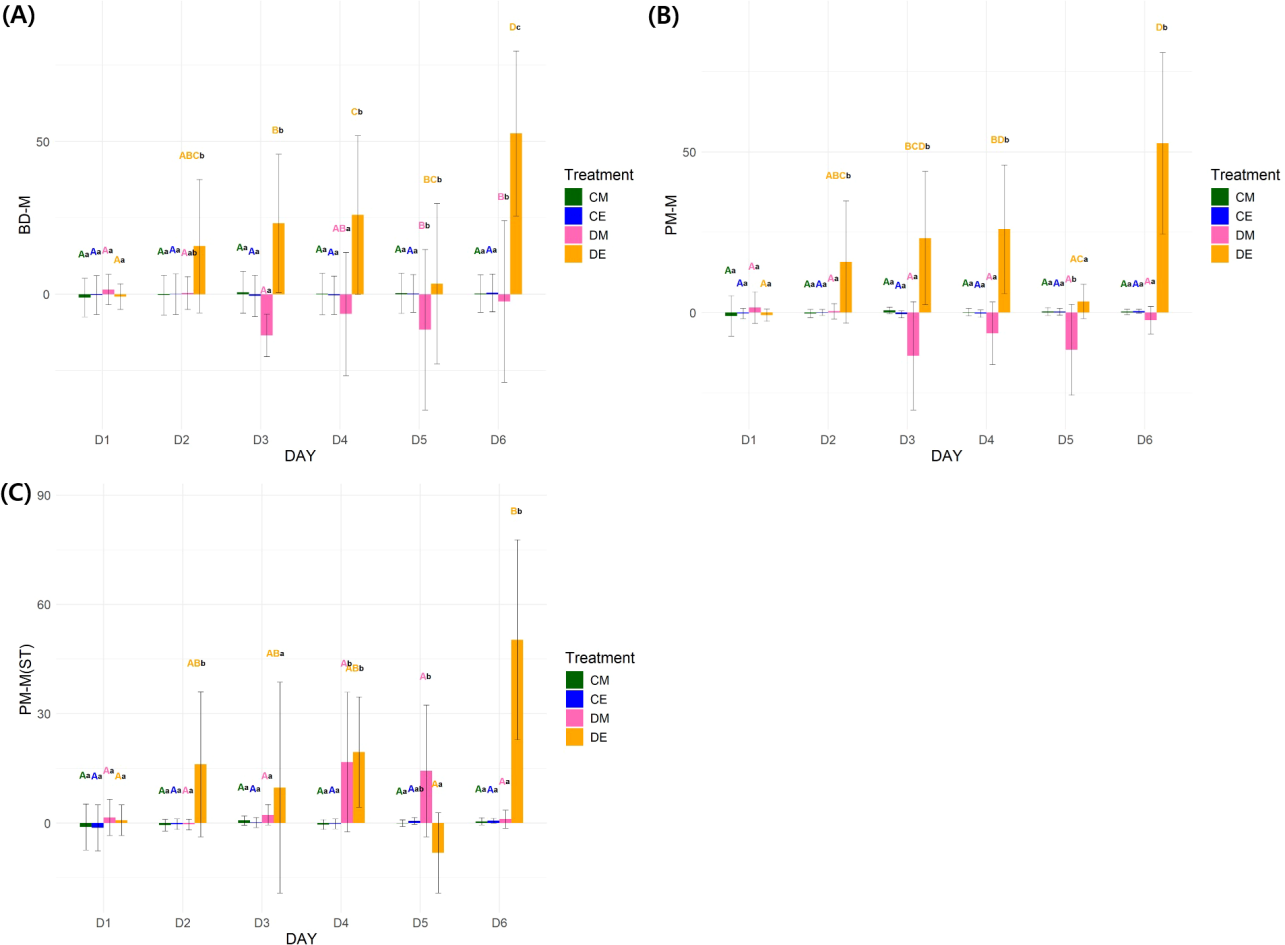
Statistical results of needle angle parameters. **(A)**: Needle angle parameter (BD-M) changes over time; **(B)**: Needle angle parameter (PM-M) changes over time; **(C)**: Needle angle parameter (PM-M(ST)) changes over time. 3-way RMANOVA was conducted, followed by pairwise t-tests with Bonferroni correction for *post-hoc* analysis (n = 30) (P < 0.05). Uppercase letters: comparison among days within each treatment; Lowercase letters: comparison among treatments within each day. BD-M: needle angle before drought treatment – current needle angle; PM-M, previous needle angle – current needle angle; PM-M(ST), previous needle angle measured at the same time of day – current needle angle; CM, control morning; DM, drought treatment morning; CE, control evening; DE, drought treatment evening.

Unlike physiological traits, needle angle parameters showed significant differences (P < 0.001) in all interaction effects (day × time × treatment) (**Supplementary Table S3**). No stress response was observed in the control on D2, as indicated by the chlorophyll fluorescence results (**Figure 6**). The DE needle angle parameters (BD-M: 16.94 ± 43.63, PM-M: 15.75 ± 38.02, PM-M(ST): 16.15 ± 39.76) exhibited a faster response to drought stress than physiological traits, with significant differences appearing from D2E. PM-M showed a shift to a negative value (-13.47 ± 33.71) on D3M, indicating signs of recovery from wilting. When monitoring needle angle changes only in the morning, significant differences were observed starting from D4M (16.76 ± 38.33) (**Figure 6C**). These results suggest that needle angle can be used as a tool for detecting drought stress earlier than physiological traits.

#### 3.3.3 Multiple regression analysis

The STx(M) and BD-M(M) models demonstrated high explanatory power (R² > 0.5) compared to the evening needle angles (**Tables 3**, **4**). The evening wilting-morning recovery cycle caused variability, leading to lower consistency across seedlings. Significant predictors for the STx(M) model included ΦNPQ, LTD, AH(P), and SR, while BD-M(M) model identified ΦNPQ, SR, AH(P), and CWSI(Tl) as significant predictors. Among the two models, ΦNPQ exhibited the highest standardized coefficient beta (> 0.5), indicating its dominant influence on needle angles. In contrast, SM was not identified as a significant predictor. Additionally, needle temperature parameters consistently emerged as significant predictors across all 8 models, highlighting their role in detecting early heat dissipation responses induced by drought stress. However, the Durbin-Watson values were close to 1 (indicating positive autocorrelation), suggesting a decrease in the independence of residuals, which could potentially affect the reliability and validity of the regression models.

**Table 3:** Stepwise multiple regression model results for morning needle angle parameters.

STx(M)	R²	adjusted R²	F Change	p-value	Durbin-Watson
	0.530	0.512	9.351	0.003	1.180
	β	standardized coefficient beta	p-value	tolerance	VIF
Intercept	9.459	–	0.000	–	–
ΦNPQ	-7.098	-0.560	0.000	0.874	1.144
LTD	0.163	0.214	0.004	0.842	1.188
AH(P)	-0.046	-0.249	0.000	0.974	1.027
SR	-0.004	-0.222	0.003	0.853	1.172
Y=9.459−7.098·ΦNPQ+0.163·LTD−0.046·AH(P)−0.004·SR					

BD-M(M)	R²	adjusted R²	F Change	p-value	Durbin-Watson
	0.529	0.511	12.252	0.001	1.189
	β	standardized coefficient beta	p-value	tolerance	VIF
Intercept	-165.030	–	0.000	–	–
ΦNPQ	215.648	0.541	0.000	0.886	1.128
SR	0.171	0.330	0.000	0.943	1.060
AH(P)	1.508	0.258	0.000	0.982	1.019
CWSI(Tl)	46.916	0.247	0.001	0.902	1.109
Y=−165.030+215.648·ΦNPQ+0.171·SR+1.508·AH(P)+46.196·CWSI(Tl)					

PM-M(M)	R²	adjusted R²	F Change	p-value	Durbin-Watson
	0.135	0.111	6.361	0.013	1.845
	β	standardized coefficient beta	p-value	tolerance	VIF
Intercept	-71.750	–	0.002	–	–
EC(P)	632.370	0.225	0.015	0.993	1.007
CWSI(Tl)	15.813	0.303	0.003	0.802	1.246
SR(P)	0.013	0.254	0.013	0.805	1.243
Y=−71.750+632.370·EC(P)+15.813·CWSI(Tl)+0.013·SR(P)					

PM-M(ST)(M)	R²	adjusted R²	F Change	p-value	Durbin-Watson
	0.356	0.344	23.162	0.000	0.811
	β	standardized coefficient beta	p-value	tolerance	VIF
Intercept	57.629	–	0.000	–	–
LTD	-22.427	-1.173	0.000	0.218	4.595
CWSI(Tl)	-121.810	-0.800	0.000	0.218	4.595
Y=57.629−22.427·LTD−121.809·CWSI(Tl)					

(P) indicates the data during the measurement times for physiological traits.

**Table 4:** Stepwise multiple regression model results for evening needle angle parameters.

STx(E)	R²	adjusted R²	F Change	p-value	Durbin-Watson
	0.391	0.374	9.213	0.003	1.108
	β	standardized coefficient beta	p-value	tolerance	VIF
Intercept	-7.125	–	0.017	–	–
LTD	0.458	0.464	0.000	0.921	1.086
EC(P)	137.030	0.324	0.000	0.955	1.047
SM	-0.074	-0.234	0.003	0.963	1.038
Y=−7.125+0.458·LTD+137.030·EC(P)−0.074·SM					

BD-M(E)	R²	adjusted R²	F Change	p-value	Durbin-Watson
	0.381	0.364	4.074	0.046	1.281
	β	standardized coefficient beta	p-value	tolerance	VIF
Intercept	406.771	–	0.000	–	–
CWSI(Tl)	102.112	0.420	0.000	0.915	1.093
EC(P)	-3811.400	-0.291	0.000	0.901	1.109
ΦII	-110.420	-0.169	0.046	0.830	1.205
Y=406.771+102.112·CWSI(Tl)−3811.433·EC(P)−110.423·ΦII					

PM-M(E)	R²	adjusted R²	F Change	p-value	Durbin-Watson
	0.314	0.288	13.665	0.000	1.105
	β	standardized coefficient beta	p-value	tolerance	VIF
Intercept	644.871	–	0.000	–	–
EC(P)	-7059.900	-0.614	0.000	0.627	1.594
SR	-4.575	-0.402	0.000	0.905	1.105
Fv'/Fm'	189.935	0.336	0.000	0.831	1.203
CWSI(Tl-Ta)	67.101	0.353	0.000	0.716	1.397
Y=644.871−7059.910·EC(P)−4.575·SR+189.935·Fv'/Fm'+67.101·CWSI(Tl-Ta)					

PM-M(ST)(E)	R²	adjusted R²	F Change	p-value	Durbin-Watson
	0.304	0.277	7.483	0.007	0.942
	β	standardized coefficient beta	p-value	tolerance	VIF
Intercept	67.055	–	0.209	–	–
CWSI(Tl)	53.752	0.263	0.003	0.878	1.139
AH(P)	-3.114	-0.495	0.000	0.668	1.498
AT	1.039	0.475	0.000	0.590	1.696
Fv'/Fm'	130.694	0.241	0.007	0.852	1.173
Y=67.055+53.752·CWSI(Tl)−3.114·AH(P)+1.039·AT+130.694·Fv'/Fm'					

(P) indicates the data during the measurement times for physiological traits.

#### 3.3.4 PCA

**Figure 7:**
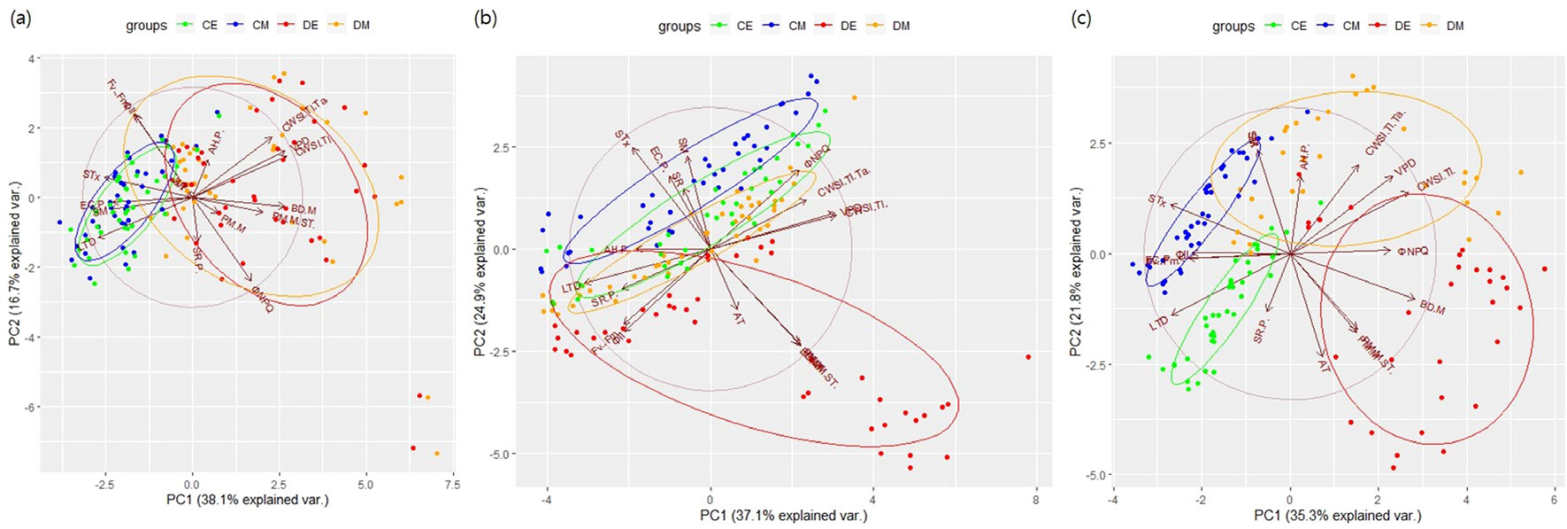
PCA results by experimental dates. **(A)**: Day 2; **(B)**: Day 4; **(C)**: Day 6.

On D2E, drought treatment formed a distinct group, with needle angle parameters showing the greatest influence (**Figure 7**). By D4, clear group separations were observed between control and drought treatments, regardless of time of day. On D6, the morning and evening groups of control were similar, whereas drought treatment groups showed distinct differences. These results indicate significant diurnal variations in apical vigor under continuous drought stress. The cumulative variance explained by PC1 and PC2 remained below 60%, suggesting complex and non-linear interactions among parameters.

### 3.4 Seedling level measurements

#### 3.4.1 Non-parametric tests

In BD-M, Longest Path and Height showed significant differences (P < 0.05) between the control and drought treatments from D4 until the end of the experiment (**Table 5**). In DE, Center of Mass(y) was able to detect drought stress from D2. In PM-M, Convex Hull Area showed significant differences (P < 0.05) among the four treatments from D4 until the end of the experiment, with differences also observed between DM and DE (**Supplementary Table S4**). Center of Mass(y) exhibited significant differences (P < 0.05) among the four treatments from D3 to D5, and differences were also observed between DM and DE. Unlike BD-M, PM-M did not show significant differences (P > 0.05) over time. In PM-M(ST), significant differences (P < 0.05) among the four treatments and the drought treatment were observed at D3 or D4, but no persistent differences were observed afterward (**Table 6**). Center of Mass(y) and Ellipse Center(y) showed significant differences over time in CE and DE (P < 0.01). Notably, Center of Mass(y) in DE showed a continuous difference from D2 to the end of the experiment compared to D1. This suggests that the wilting of the apical part in the evening also affected seedling level parameters. Overall, distinct trends were observed depending on the needle angle parameter, and Center of Mass(y), which reflects changes in seedling height, was identified as the most suitable seedling level parameter for detecting drought stress.

**Table 5:** Statistical results of seedling level image analysis (BD-M).

BD-M		CM	CE	DM	DE
Longest Path	D1	-69.10 ± 156.27	-13.00 ± 141.03	247.60 ± 846.41	-33.50 ± 92.34
	D2	-5.80 ± 153.44	-67.50 ± 110.00	-31.50 ± 190.75	113.60 ± 393.45
	D3	-45.90 ± 138.50	-62.80 ± 110.08	43.89 ± 291.61	328.67 ± 502.24
	D4	*-106.00 ± 156.72a*	*-133.30 ± 155.91a*	*386.67 ± 530.06b*	*399.22 ± 523.86b*
	D5	*-4.80 ± 364.68a*	*-129.00 ± 103.53a*	*383.00 ± 482.44b*	*435.86 ± 489.68ab*
	D6	*-76.70 ± 104.80a*	*-145.90 ± 121.58a*	*299.14 ± 531.82b*	*412.00 ± 477.20ab*
Height	D1	-4.80 ± 21.25	3.80 ± 18.22	5.40 ± 16.39	2.50 ± 12.82
	D2	-0.90 ± 26.11	-7.50 ± 16.93	-4.40 ± 24.88	23.90 ± 52.67
	D3	-3.60 ± 17.88	-4.70 ± 14.55	8.00 ± 41.67	49.00 ± 74.41
	D4	*-10.20 ± 25.05a*	*-13.70 ± 19.87a*	*50.89 ± 77.54ab*	*63.33 ± 78.88b*
	D5	*-15.50 ± 21.86a*	*-13.80 ± 12.83ab*	*50.00 ± 80.41b*	*64.43 ± 76.14b*
	D6	*-13.20 ± 18.70a*	*-21.50 ± 16.81ab*	*53.00 ± 76.68b*	*60.71 ± 72.18b*
Perimeter	D1	451.44 ± 1963.78	-343.53 ± 1595.32	1288.96 ± 1806.91	-31.81 ± 894.03
	D2	207.69 ± 1145.07	-455.38 ± 1058.74	1343.25 ± 1417.81	1445.47 ± 1906.48
	D3	*-351.32 ± 765.64a*	*-756.84 ± 1149.22a*	*1491.84 ± 1770.05b*	*1488.67 ± 2503.51ab*
	D4	1016.40 ± 1465.51	1106.16 ± 1904.31	3087.52 ± 3134.50	4365.14 ± 4949.77
	D5	741.16 ± 1100.04	-373.51 ± 1737.12	2658.95 ± 2585.94	2565.46 ± 3295.65
	D6	546.15 ± 1397.76	74.54 ± 1289.52	2658.01 ± 2618.84	2729.93 ± 2467.69
Center of Mass(y)	D1	2.72 ± 9.60	2.10 ± 9.42	1.17 ± 7.07	**-0.96 ± 9.64A**
	D2	-4.72 ± 8.62	-0.73 ± 9.23	-3.87 ± 11.25	**-25.36 ± 33.00AB**
	D3	-0.29 ± 7.71	5.15 ± 7.54	-6.72 ± 37.40	**-39.57 ± 35.88B**
	D4	-1.84 ± 8.37	-0.74 ± 8.81	7.29 ± 10.26	**-47.87 ± 42.86B**
	D5	*1.92 ± 4.31a*	*3.25 ± 14.81a*	*8.06 ± 10.43ab*	***-47.95 ± 40.26Bb***
	D6	-3.09 ± 9.86	-2.95 ± 10.99	3.33 ± 15.24	**-51.50 ± 41.39B**

Mean ± SD. Kruskal-Wallis test was conducted, followed by Mann-Whitney test with Bonferroni correction for *post-hoc* analysis (P < 0.05). Uppercase letters: comparison among days within each treatment; Lowercase letters: comparison among treatments within each day; **Bold letters**: significant difference among days within each treatment; *Italic letters*: significant difference among treatments within each day.

**Table 6:** Statistical results of seedling level image analysis (PM-M(ST)).

PM-M(ST)		CM	CE	DM	DE
Height	D1	-4.80 ± 21.25	3.80 ± 18.22	5.40 ± 16.39	5.72 ± 1.76
	D2	3.90 ± 13.43	-11.30 ± 13.40	-9.80 ± 31.72	21.40 ± 45.51
	D3	-2.70 ± 12.94	2.80 ± 8.35	16.00 ± 36.19	38.33 ± 52.87
	D4	*-6.60 ± 11.24a*	*-9.00 ± 13.94a*	*42.89 ± 60.03ab*	*14.33 ± 19.83b*
	D5	-5.30 ± 10.56	-0.10 ± 14.79	0.43 ± 9.29	0.71 ± 12.08
	D6	2.30 ± 13.57	-7.70 ± 10.86	3.00 ± 15.36	-3.71 ± 12.94
Center of Mass(y)	D1	*-3.91 ± 9.14a*	***-1.81 ± 6.68ABa***	*1.17 ± 7.07ab*	***5.62 ± 1.23Ab***
	D2	-2.62 ± 8.39	**-5.45 ± 7.76A**	-6.01 ± 7.39	**-24.40 ± 37.07B**
	D3	*-1.02 ± 8.69a*	***4.86 ± 8.67Ba***	*-11.28 ± 32.55ab*	***-19.57 ± 16.87Bb***
	D4	3.31 ± 9.15	**-2.58 ± 7.20AB**	-17.51 ± 30.47	**-8.31 ± 15.25AB**
	D5	1.18 ± 12.11	**5.17 ± 12.77AB**	-7.47 ± 14.65	**-2.20 ± 9.94AB**
	D6	0.16 ± 8.76Aa	**-6.04 ± 6.76A**	-6.93 ± 16.85	**-3.54 ± 7.95AB**
Eillpse Center(y)	D1	*-2.66 ± 7.06a*	***-0.55 ± 6.63ABab***	*-0.77 ± 11.81ab*	***5.57 ± 1.23Ab***
	D2	-0.96 ± 8.35	**-8.34 ± 12.16A**	-3.93 ± 8.84	**-22.79 ± 31.66B**
	D3	*-0.56 ± 10.84ab*	***5.83 ± 8.33Ba***	*-8.51 ± 27.34ab*	***-19.71 ± 20.49Bb***
	D4	1.00 ± 10.66	**-2.56 ± 10.45AB**	-23.54 ± 30.31	**-6.04 ± 16.60AB**
	D5	-0.46 ± 11.67	**6.77 ± 10.46B**	-7.83 ± 15.78	**-2.36 ± 9.15AB**
	D6	-1.45 ± 9.86	**-8.07 ± 9.49A**	-1.94 ± 13.78	**-0.69 ± 9.41AB**
Longest Path	D1	-69.10 ± 156.27	-13.00 ± 141.03	247.60 ± 846.41	40.30 ± 11.73
	D2	63.30 ± 140.21	-54.50 ± 100.68	-279.10 ± 835.71	147.10 ± 343.38
	D3	-40.10 ± 111.52	4.70 ± 64.16	88.00 ± 231.29	316.11 ± 355.15
	D4	*-60.10 ± 57.25a*	*-70.50 ± 115.48a*	*342.78 ± 401.00b*	*70.56 ± 98.70b*
	D5	101.20 ± 400.16	4.30 ± 113.06	0.14 ± 118.16	44.71 ± 133.49
	D6	-71.90 ± 417.35	-16.90 ± 62.27	-83.86 ± 148.22	-23.86 ± 119.91

Mean ± SD. Kruskal-Wallis test was conducted, followed by Mann-Whitney test with Bonferroni correction for *post-hoc* analysis (P < 0.05). Uppercase letters: comparison among days within each treatment; Lowercase letters: comparison among treatments within each day; **Bold letters**: significant difference among days within each treatment; *Italic letters*: significant difference among treatments within each day.

#### 3.4.2 Multiple regression analysis

Unlike the RMANOVA results, no significant predictors were identified in DE for constructing a regression model. In contrast, a regression model was successfully constructed in DM, but its R² values remained below 0.3, considerably lower than those of the needle angles (**Table 7**). SR was identified as a significant predictor in both the Height and Center of Mass(y) models. Likewise, SR also emerged as a significant predictor in the apical needle angle-based STx(M) and BD-M(M) models, highlighting its sensitive influence on needle angles under drought stress (**Table 3**).

**Table 7:** Stepwise multiple regression model results for seedling level image analysis.

STx(M)	R²	adjusted R²	F Change	p-value	Durbin-Watson
	0.261	0.230	8.466	0.008	1.930
	β	standardized coefficient beta	p-value	tolerance	VIF
Intercept	-45.001	–	0.043	–	–
SR	0.348	0.511	0.008	1.000	1.000
Y=−45.001+0.348·SR					

BD-M(M)	R²	adjusted R²	F Change	p-value	Durbin-Watson
	0.294	0.233	4.359	0.048	1.952
	β	standardized coefficient beta	p-value	tolerance	VIF
Intercept	-41.528	–	0.001	–	–
SPAD	1.358	0.439	0.020	0.989	1.011
SR(P)	0.038	0.368	0.048	0.989	1.011
Y=−41.528+1.358·SPAD+0.038·SR(P)					

(P) indicates the data during the measurement times for physiological traits.

## 4 Discussion

### 4.1 Physiological traits

Physiological measurements successfully identified drought stress non-destructively in seedlings before visible needle discoloration. The study results demonstrated that different physiological parameters vary in their effectiveness in detecting specific stress conditions. Chlorophyll fluorescence showed particularly sensitive to light and heat stress in the control, likely due to exposure to high light conditions. Over time, the drought treatment exhibited a stress response due to the absence of irrigation, whereas no such response was observed in the control.

Heat stress damages the protein structure of PSII complexes and directly affects the structural stability of the photosynthetic mechanism, leading to a sharp response in chlorophyll fluorescence (Allakhverdiev et al., 2008). Excessive light also induces the formation of reactive oxygen species (ROS), leading to PSII damage, as evidenced by higher Fm’ and Fo’ values in the control compared to the drought treatment and an increase in ΦNPQ in the drought treatment on D2 (Murata et al., 2007). Previous studies have reported Fv/Fm decreases under heat stress in various crop species, highlighting the sensitivity of chlorophyll fluorescence to heat stress (Guo et al., 2006; Carmo-Silva and Salvucci, 2012; Barboričová et al., 2022).

In contrast, mild drought stress has been reported to have minimal impact on Fv/Fm, suggesting that chlorophyll fluorescence parameters may exhibit a delayed response under short-term drought conditions when soil moisture is still available (Baker and Rosenqvist, 2004). This aligns with the study results, where the main effect was observed over time rather than between treatments, as drought stress did not appear immediately.

Needle temperature parameters showed no indication of light or heat stress in the control. Meanwhile, VPD calculated with air temperature as a factor was lower on D2 than on D4 in the drought treatment, indicating the influence of high temperatures that day. During drought conditions, plants regulate stomatal activity to maintain internal water levels, making them relatively less sensitive to light and heat stress (Chaves et al., 2009; Martínez-Vilalta and Garcia-Forner, 2017). The structural traits of *L. kaempferi* needles, such as stomatal area and density, reduce their efficiency in heat dissipation through transpiration compared to broadleaf species. This is consistent with LTD showing a rapid stress response on D4. Therefore, thermal imaging holds great potential for detecting drought stress and improving irrigation management in *L. kaempferi* nurseries.

EC also emerged as the earliest physiological response to drought stress, consistent with water potential and osmotic effects reported in previous studies (Yang et al., 2016). Park et al. (2019) noted that dynamic environmental conditions, such as cloudy days, were reflected in EC measurements. This suggests that EC could be a valuable tool for real-time monitoring of drought stress and other unusual cultivation conditions. However, direct irrigation in soil may have led to over- or underestimation of drought stress, highlighting the need for further studies using sprinkler irrigation to enhance accuracy.

### 4.2 Comparative analysis with needle angle-based phenotypes

Image-based measurements of apical vigor allowed earlier drought stress detection compared to physiological traits. The apical part, positioned at the seedling tip, is the last to receive water through xylem transport and is structurally and physiologically more sensitive to water deficits. Apical needles are thinner, more vulnerable to turgor loss, and exposed to direct sunlight, which accelerates wilting under drought stress (Gebauer et al., 2015; Nadal et al., 2020; Jahan et al., 2023). Stepwise multiple regression analysis confirmed that SR was the most influential factor for both apical needle angles and seedling level parameters. Although the study results did not show an immediate stress response to strong SR in needle angles, its impact was evident in the needle angle RMANOVA results through the significant interaction between day and treatment. Changes in needle angles reflect physiological adaptation strategies to optimize photosynthesis while minimizing heat stress (Nilsen and Forseth, 2018). Under drought conditions, heat and light stress synergistically exacerbate damage, leading to rapid needle wilting in *L. kaempferi* (Kim et al., 2022). This aligns with findings suggesting that shading treatments can mitigate drought-induced damage and that this species is more susceptible to high temperatures than drought (Kim et al., 2024). Thus, when drought stress is induced by the absence of irrigation, SR becomes a key factor in monitoring needle angle changes, particularly when drought period (day and time) is considered as a variable. This suggests its importance in determining the duration of needle angle recovery and maintaining stability, as well as optimizing irrigation timing.

### 4.3 Applicability of image analysis methods at seedling level

As mentioned in the introduction, while real-time leaf angle analysis systems have been developed, they are often species-specific and require further optimization for *L. kaempferi*. The continuous growth pattern of needles along the stem poses challenges in distinguishing the apical part through imaging. In particular, the young stems of *L. kaempferi*, being less lignified, may show structural irregularities that further complicate analysis. Therefore, seedling level image analysis improved practicality by providing objective parameters for seedling morphology that cannot be measured manually. Furthermore, expanding the analysis from the seedling level to the container level could allow for image-based assessment of seedlings within a single area, enabling its application for drought stress diagnosis.

The results of this study showed that changes in the height (y-axis) of *L. kaempferi* significantly affect the canopy shape in images. This suggests a broad applicability for simultaneously monitoring growth parameters such as height growth. Additionally, since *L. kaempferi* is sensitive to heat, integrating thermal imaging could help identify irrigation blind spots in greenhouse environments, facilitating the development of a dynamic and efficient irrigation management system. Such imaging systems are expected to effectively analyze seedling growth and physiological status, contributing to the production of high-quality *L. kaempferi*.

However, measuring with 2D-RGB images alone may lack precision, highlighting the need to explore cost-effective solutions to address this limitation. Color correction issues may arise due to variations in light intensity, potentially leading to inconsistencies in color recognition even under the same stress conditions. Therefore, it is necessary to refine the standard light source calibration for color correction or develop analytical images in a way that minimizes errors. As a method to enhance camera performance, Liu et al. (2021) introduced a real-time, precise stress measurement technique at the individual plant level using RGB-D (depth) imaging. This approach was used to assess plant height and aboveground biomass in *Toona sinensis* seedlings subjected to drought stress.

The leaf projection function (G function) have been introduced to evaluate the spatial arrangement of leaves, analyze light environments, and model the structural characteristics of plants, enabling a detailed analysis of plant-light interactions (Pisek et al., 2011; Liu et al., 2021). Integrating the G function into a smart nursery system would enable the adjustment of appropriate spacing between seedlings and leaf arrangement, enhancing light utilization and facilitating the design of an optimal tree structure.

Meanwhile, in this study, although the apical part did not wither, partial canopy dieback or leaf abscission was observed. A rapid decrease in needle has been reported as a key factor accelerating the forest dieback process (Sangüesa-Barreda et al., 2023). The needle trace method (NTM) is a technique that quantifies the dynamics of needle production by analyzing cross-sections or longitudinal sections of the stem based on traces of needle attachment (Pouttu and Dobbertin, 2000; Drenkhan et al., 2006; Sangüesa-Barreda et al., 2023). Similarly, an image analysis technique for detecting partial canopy dieback or leaf abscission could serve as a drought tolerance assessment parameter for the production of high-quality seedlings. Furthermore, this technique is expected to function as a critical monitoring tool for adjusting the intensity of irrigation and priming treatments.

By integrating and optimizing various methods, early detection of drought stress at the seedling level can be achieved while also facilitating diverse tree physiological analyses, supporting the cultivation of high-quality seedlings.

### 4.4 Limitations and future studies

In this study, while the correlation between physiological traits and needle angles was analyzed, difficulties arose in perfectly synchronizing physiological data and needle angle measurements at the same seedling and time point. Addressing this issue will be essential for achieving more precise analyses in the future. Additionally, direct irrigation was applied to the control soil instead of using sprinklers, which excluded factors such as environmental conditions or dew formation on the needle that could influence needle angles. For seedling level analysis, an adequate sample size of surviving seedlings is necessary to ensure sufficient statistical power for parametric tests. Therefore, future studies should aim to develop experiments in large-scale nursery settings to improve practical applicability. Moreover, the period during which apical vigor recovers and seedling survival stabilizes may vary depending on weather conditions during the growing season or individual plant characteristics. This highlights the need for additional research to determine whether growth is sustained after apical wilting and recovery at different stages of the growing season. If growth continues, it could facilitate more efficient irrigation strategies while also leveraging the priming effects induced by stress treatments, further optimizing seedling resilience and resource management.

## 5 Conclusion

This study demonstrated the potential of needle angle-based phenotypic measurements for the early detection of drought stress in *L. kaempferi* seedlings. Overall, tree physiological traits detected drought stress on D6. Chlorophyll fluorescence effectively detected light and heat stress, whereas needle temperature parameters were more sensitive to drought stress. Needle angles provided the earliest indicators of drought stress, with significant changes observed by D2, even before physiological symptoms were evident. Apical needle wilting occurred in the evening and recovered the following morning, with most seedlings showing mortality within 1–2 days. Seedling level image analysis validated these findings, Center of Mass(y) as significant parameter for drought stress monitoring. Combining apical and seedling level parameters enhances the applicability of image-based methods for stress detection. These methods can be further integrated with thermal imaging to optimize irrigation systems and monitor seedling growth dynamically. Future advancements in imaging techniques are expected to enhance precision and broaden the scope of phenotypic measurements in nursery systems. This study lays the groundwork for developing efficient tools for monitoring drought stress, combining physiological and phenotypic approaches, to support the production of resilient, high-quality *L. kaempferi* seedlings.

## Data Availability

The original contributions presented in the study are included in the article/**Supplementary Material**. Further inquiries can be directed to the corresponding authors.
