# Supplementary material for: Needle angle dynamics as a rapid indicator of drought stress in Larix kaempferi (Lamb.) Carrière: advancing non-destructive imaging techniques for resilient seedling production
Source: Front Plant Sci. 2025 May 12;16:1550748. doi: 10.3389/fpls.2025.1550748 (PMC12104679; doi:10.3389/fpls.2025.1550748)
Supplement: Supplementary file 1 [file Table1.docx]

**Supplementary Table 1.** Results of 2-way RMANOVA for physiological traits (n = 30).

|  | | Sum of Square | df | Mean Square | F | P |
| --- | --- | --- | --- | --- | --- | --- |
| Day | Fm' | 8093507.066 | 1.980 | 4087045.871 | 3.144 | 0.047 |
|  | Fo' | 1110515.544 | 1.903 | 583582.398 | 7.736 | 0.001 |
|  | Fv'/Fm' | 0.055 | 1.496 | 0.037 | 6.266 | 0.006 |
|  | ΦII | 0.057 | 1.426 | 0.040 | 6.638 | 0.006 |
|  | ΦNO | 0.004 | 1.910 | 0.002 | 3.172 | 0.048 |
|  | ΦNPQ | 0.091 | 1.493 | 0.061 | 6.391 | 0.006 |
|  | PSIact | 21.791 | 1.182 | 23.505 | 1.188 | 0.289 |
|  | PSIopen | 0.012 | 1.752 | 0.007 | 0.010 | 0.983 |
|  | qL | 0.001 | 1.974 | 0.001 | 0.126 | 0.880 |
|  | SPAD | 371.253 | 1.988 | 186.719 | 1.727 | 0.183 |
|  | EC | 0.003 | 0.000 | 0.000 | 0.000 | 0.000 |
|  | VPD | 287.369 | 1.618 | 177.588 | 414.041 | 0.000 |
|  | CWSI(Tl) | 0.529 | 1.380 | 0.383 | 8.735 | 0.002 |
|  | CWSI(Tl-Ta) | 0.235 | 1.536 | 0.153 | 2.316 | 0.117 |
|  | LTD | 41.825 | 1.527 | 27.388 | 12.334 | 0.000 |
| Treatment | Fm' | 87722799.219 | 1 | 87722799.219 | 110.961 | 0.000 |
|  | Fo' | 5357505.089 | 1 | 5357505.089 | 130.675 | 0.000 |
|  | Fv'/Fm' | 0.013 | 1 | 0.013 | 3.437 | 0.069 |
|  | ΦII | 0.005 | 1 | 0.005 | 1.446 | 0.234 |
|  | ΦNO | 0.003 | 1 | 0.003 | 4.713 | 0.034 |
|  | ΦNPQ | 0.015 | 1 | 0.015 | 2.566 | 0.115 |
|  | PSIact | 3.981 | 1 | 3.981 | 0.361 | 0.551 |
|  | PSIopen | 0.249 | 1 | 0.249 | 0.422 | 0.519 |
|  | qL | 0.025 | 1 | 0.025 | 4.412 | 0.050 |
|  | SPAD | 178.205 | 1 | 178.205 | 3.244 | 0.077 |
|  | EC | 0.038 | 1 | 0.038 | 0.000 | 0.000 |
|  | VPD | 9.758 | 1 | 9.758 | 11.198 | 0.001 |
|  | CWSI(Tl) | 2.247 | 1 | 2.247 | 22.940 | 0.000 |
|  | CWSI(Tl-Ta) | 1.218 | 1 | 1.218 | 8.575 | 0.005 |
|  | LTD | 73.225 | 1 | 73.225 | 15.398 | 0.000 |
| Day  ×  Treatment | Fm' | 18864244.654 | 1.980 | 9526035.203 | 7.327 | 0.001 |
|  | Fo' | 265172.678 | 1.903 | 139349.789 | 1.847 | 0.164 |
|  | Fv'/Fm' | 0.134 | 1.496 | 0.067 | 15.226 | 0.000 |
|  | ΦII | 0.147 | 1.426 | 0.103 | 17.123 | 0.000 |
|  | ΦNO | 0.009 | 1.910 | 0.005 | 7.171 | 0.001 |
|  | ΦNPQ | 0.227 | 1.493 | 0.152 | 15.969 | 0.000 |
|  | PSIact | 45.484 | 1.182 | 38.468 | 1.944 | 0.166 |
|  | PSIopen | 3.507 | 1.752 | 2.002 | 2.945 | 0.064 |
|  | qL | 0.006 | 1.974 | 0.003 | 0.585 | 0.557 |
|  | SPAD | 129.920 | 1.988 | 65.343 | 0.604 | 0.547 |
|  | EC | 0.004 | 0.000 | 0.000 | 0.000 | 0.000 |
|  | VPD | 7.450 | 1.618 | 4.604 | 10.734 | 0.000 |
|  | CWSI(Tl) | 1.445 | 1.380 | 1.047 | 23.886 | 0.000 |
|  | CWSI(Tl-Ta) | 0.579 | 1.536 | 0.377 | 5.710 | 0.009 |
|  | LTD | 51.694 | 1.527 | 33.850 | 15.244 | 0.000 |

Post-hoc analysis was carried out using pairwise t-tests with Bonferroni correction (P < 0.05). The Greenhouse-Geisser correction was applied for sphericity. Day: D1–D6; Treatment: control, drought, Time: morning, evening.
