# Supplementary material for: Needle angle dynamics as a rapid indicator of drought stress in Larix kaempferi (Lamb.) Carrière: advancing non-destructive imaging techniques for resilient seedling production
Source: Front Plant Sci. 2025 May 12;16:1550748. doi: 10.3389/fpls.2025.1550748 (PMC12104679; doi:10.3389/fpls.2025.1550748)
Supplement: Supplementary file 2 [file Table2.docx]

**Supplementary Table 2.** Results of t-test by day and treatment.

|  | Inter-day | | P | Inter-treatment | | P |
| --- | --- | --- | --- | --- | --- | --- |
| Fm' | Control | D2-D4 | 0.924 | Day 2 | C-D | 0.002 |
|  |  | D2-D6 | 0.001 |  |  |  |
|  |  | D4-D6 | 0.001 | Day 4 | C-D | 0.000 |
|  | Drought | D2-D4 | 0.812 |  |  |  |
|  |  | D2-D6 | 0.234 | Day 6 | C-D | 0.000 |
|  |  | D4-D6 | 0.374 |  |  |  |
| Fo' | Control | D2-D4 | 0.085 | Day 2 | C-D | 0.000 |
|  |  | D2-D6 | 0.059 |  |  |  |
|  |  | D4-D6 | 0.000 | Day 4 | C-D | 0.000 |
|  | Drought | D2-D4 | 0.619 |  |  |  |
|  |  | D2-D6 | 0.151 | Day 6 | C-D | 0.000 |
|  |  | D4-D6 | 0.055 |  |  |  |
| Fv'/Fm' | Control | D2-D4 | 0.001 | Day 2 | C-D | - |
|  |  | D2-D6 | 0.000 |  |  |  |
|  |  | D4-D6 | 0.741 | Day 4 | C-D |  |
|  | Drought | D2-D4 | 0.806 |  |  |  |
|  |  | D2-D6 | 0.002 | Day 6 | C-D |  |
|  |  | D4-D6 | 0.001 |  |  |  |
| ΦII | Control | D2-D4 | 0.000 | Day 2 | C-D | - |
|  |  | D2-D6 | 0.000 |  |  |  |
|  |  | D4-D6 | 0.840 | Day 4 | C-D |  |
|  | Drought | D2-D4 | 0.823 |  |  |  |
|  |  | D2-D6 | 0.001 | Day 6 | C-D |  |
|  |  | D4-D6 | 0.001 |  |  |  |
| ΦNO | Control | D2-D4 | 0.118 | Day 2 | C-D | 0.362 |
|  |  | D2-D6 | 0.062 |  |  |  |
|  |  | D4-D6 | 0.702 | Day 4 | C-D | 0.712 |
|  | Drought | D2-D4 | 0.584 |  |  |  |
|  |  | D2-D6 | 0.012 | Day 6 | C-D | 0.002 |
|  |  | D4-D6 | 0.006 |  |  |  |
| ΦNPQ | Control | D2-D4 | 0.000 | Day 2 | C-D | - |
|  |  | D2-D6 | 0.000 |  |  |  |
|  |  | D4-D6 | 0.947 | Day 4 | C-D |  |
|  | Drought | D2-D4 | 0.943 |  |  |  |
|  |  | D2-D6 | 0.001 | Day 6 | C-D |  |
|  |  | D4-D6 | 0.001 |  |  |  |
| EC | Control | D2-D4 | 0.000 | Day 2 | C-D | 0.000 |
|  |  | D2-D6 | 0.000 |  |  |  |
|  |  | D4-D6 | 0.000 | Day 4 | C-D | 0.000 |
|  | Drought | D2-D4 | 0.000 |  |  |  |
|  |  | D2-D6 | 0.000 | Day 6 | C-D | 0.000 |
|  |  | D4-D6 | 0.000 |  |  |  |
| VPD | Control | D2-D4 | 0.642 | Day 2 | C-D | 0.864 |
|  |  | D2-D6 | 0.000 |  |  |  |
|  |  | D4-D6 | 0.000 | Day 4 | C-D | 0.000 |
|  | Drought | D2-D4 | 0.000 |  |  |  |
|  |  | D2-D6 | 0.000 | Day 6 | C-D | 0.000 |
|  |  | D4-D6 | 0.000 |  |  |  |
| CWSI  (Tl) | Control | D2-D4 | 0.000 | Day 2 | C-D | 0.772 |
|  |  | D2-D6 | 0.008 |  |  |  |
|  |  | D4-D6 | 0.149 | Day 4 | C-D | 0.000 |
|  | Drought | D2-D4 | 0.350 |  |  |  |
|  |  | D2-D6 | 0.000 | Day 6 | C-D | 0.000 |
|  |  | D4-D6 | 0.000 |  |  |  |
| CWSI  (Tl-Ta) | Control | D2-D4 | - | Day 2 | C-D | 0.941 |
|  |  | D2-D6 |  |  |  |  |
|  |  | D4-D6 |  | Day 4 | C-D | 0.001 |
|  | Drought | D2-D4 |  |  |  |  |
|  |  | D2-D6 |  | Day 6 | C-D | 0.000 |
|  |  | D4-D6 |  |  |  |  |
| LTD | Control | D2-D4 | 0.630 | Day 2 | C-D | 0.751 |
|  |  | D2-D6 | 0.882 |  |  |  |
|  |  | D4-D6 | 0.294 | Day 4 | C-D | 0.000 |
|  | Drought | D2-D4 | 0.000 |  |  |  |
|  |  | D2-D6 | 0.000 | Day 6 | C-D | 0.000 |
|  |  | D4-D6 | 0.268 |  |  |  |

The p-values were adjusted using the Bonferroni correction (P < 0.05). Parameters without significant statistical results of 2-way RMANOVA were excluded.
