# Supplementary material for: Needle angle dynamics as a rapid indicator of drought stress in Larix kaempferi (Lamb.) Carrière: advancing non-destructive imaging techniques for resilient seedling production
Source: Front Plant Sci. 2025 May 12;16:1550748. doi: 10.3389/fpls.2025.1550748 (PMC12104679; doi:10.3389/fpls.2025.1550748)
Supplement: Supplementary file 3 [file Table3.docx]

**Supplementary Table 3.** Results of 3-way RMANOVA for needle angle parameters. (n = 30)

|  | | Sum of Square | df | Mean Square | F | P |
| --- | --- | --- | --- | --- | --- | --- |
| Day | BD-M | 75747.823 | 2.750 | 27541.311 | 30.489 | 0.000 |
|  | PM-M | 15662.008 | 3.318 | 4720.180 | 6.564 | 0.000 |
|  | PM-M(ST) | 14643.399 | 3.203 | 4571.991 | 4.999 | 0.002 |
| Treatment | BD-M | 132399.198 | 1 | 132399.198 | 44.558 | 0.000 |
|  | PM-M | 9836.822 | 1 | 9836.822 | 30.778 | 0.000 |
|  | PM-M(ST) | 19590.756 | 1 | 19590.756 | 78.123 | 0.000 |
| Time | BD-M | 17971.849 | 1 | 17971.849 | 5.958 | 0.016 |
|  | PM-M | 28833.807 | 1 | 28833.807 | 90.216 | 0.000 |
|  | PM-M(ST) | 3528.688 | 1 | 3528.688 | 14.071 | 0.000 |
| Day  ×  Treatment | BD-M | 72789.210 | 2.750 | 26465.583 | 29.298 | 0.000 |
|  | PM-M | 14861.551 | 3.318 | 4478.941 | 6.229 | 0.000 |
|  | PM-M(ST) | 12928.329 | 3.203 | 4036.509 | 4.414 | 0.004 |
| Day  ×  Time | BD-M | 13959.881 | 2.750 | 5075.703 | 5.619 | 0.001 |
|  | PM-M | 14745.825 | 3.318 | 4444.063 | 6.180 | 0.000 |
|  | PM-M(ST) | 20921.368 | 3.203 | 6532.111 | 7.143 | 0.000 |
| Treatment  ×  Time | BD-M | 18193.953 | 1 | 18193.953 | 6.032 | 0.016 |
|  | PM-M | 29100.665 | 1 | 29100.665 | 91.051 | 0.000 |
|  | PM-M(ST) | 3409.921 | 1 | 3409.921 | 13.598 | 0.000 |
| Day  ×  Treatment  ×  Time | BD-M | 13607.762 | 2.750 | 4947.675 | 5.477 | 0.002 |
|  | PM-M | 15799.628 | 3.318 | 4761.656 | 6.622 | 0.000 |
|  | PM-M(ST) | 21131.683 | 3.203 | 6597.776 | 7.215 | 0.000 |

Post-hoc analysis was carried out using pairwise t-tests with Bonferroni correction (P < 0.05). The Greenhouse-Geisser correction was applied for sphericity. Day: D1–D6; Treatment: control, drought, Time: morning, evening.
