# Supplementary material for: Needle angle dynamics as a rapid indicator of drought stress in Larix kaempferi (Lamb.) Carrière: advancing non-destructive imaging techniques for resilient seedling production
Source: Front Plant Sci. 2025 May 12;16:1550748. doi: 10.3389/fpls.2025.1550748 (PMC12104679; doi:10.3389/fpls.2025.1550748)
Supplement: Supplementary file 4 [file Table4.docx]

**Supplementary Table 4.** Statistical results of seedling level image analysis (PM-M).

| PM-M | | CM | CE | DM | DE |
| --- | --- | --- | --- | --- | --- |
| Area | D1 | 473.10±7554.69 | 1147.40±6246.95 | -3054.90±7482.33 | 2579.20±4927.46 |
|  | D2 | -981.60±7388.80 | -610.40±6595.65 | -4241.90±10504.28 | 7052.90±12183.94 |
|  | D3 | 961.00±6109.49 | 2830.90±6820.39 | 744.44±9001.04 | 14951.78±16217.75 |
|  | D4 | *-4255.80±5620.01ab* | *-3086.40±4563.14ab* | *-6264.67±4672.69a* | *3443.89±8102.91b* |
|  | D5 | 707.00±7867.16 | 2613.60±6023.17 | -3505.43±7884.87 | 6172.29±6370.63 |
|  | D6 | *-560.20±6358.94ab* | *-3905.80±4226.42a* | *-5340.00±4292.35a* | *4541.43±5422.43b* |
| Convex  Hull  Area | D1 | 1733.30±6050.59 | 2167.75±7403.96 | 1862.90±11347.33 | -2114.10±7612.94 |
|  | D2 | -1646.05±8116.53 | -731.15±6240.84 | -845.35±6102.13 | 6921.60±19038.68 |
|  | D3 | 542.2±5620.33 | 2660.70±6975.43 | 842.67±8261.59 | 17803.67±23394.04 |
|  | D4 | *-3094.10±5620.47a* | *-267.00±3738.72ab* | *-1992.94±6874.50ab* | *12457.61±17717.47b* |
|  | D5 | *-1459.40±4512.34ab* | *770.25±4109.37ab* | *-6751.14±8163.11a* | *5058.86±6155.54b* |
|  | D6 | *-1413.30±4635.13ab* | *-2149.40±2616.50a* | *-6117.14±5369.39a* | *5116.07±4296.86b* |
| Height | D1 | -0.40±22.29 | 8.60±16.91 | 5.40±16.39 | -2.90±20.26 |
|  | D2 | *-4.70±21.79ab* | *-6.60±19.30ab* | *-6.90±15.04a* | *28.30±40.40b* |
|  | D3 | 3.90±7.65 | -1.10±9.23 | 8.11±48.47 | 41.00±60.18 |
|  | D4 | -5.50±16.91 | -3.50±7.23 | 1.89±8.31 | 12.44±22.31 |
|  | D5 | -1.80±6.48 | 1.70±16.51 | -13.71±20.90 | 14.43±16.09 |
|  | D6 | *0.60±8.90ab* | *-8.30±7.97a* | *-11.43±12.39ab* | *7.71±11.27b* |
| Center of  Mass(y) | D1 | 2.72±9.6 | 2.10±9.42 | 1.17±7.07 | -2.14±7.89 |
|  | D2 | -4.72±8.62 | -0.73±9.23 | -3.87±11.25 | -20.52±32.04 |
|  | D3 | *-0.29±7.71ab* | *5.15±7.54a* | *-6.72±37.40ab* | *-24.80±25.46b* |
|  | D4 | *-1.84±8.37ab* | *-0.74±8.81a* | *7.29±10.26a* | *-15.59±15.75b* |
|  | D5 | *1.92±4.31ab* | *3.25±14.81ab* | *8.06±10.43a* | *-10.26±12.67b* |
|  | D6 | -3.09±9.86 | -2.95±10.99 | 3.33±15.24 | -6.87±15.92 |

Mean ± SD. Kruskal-Wallis test was conducted, followed by Mann-Whitney test with Bonferroni correction for post-hoc analysis (P < 0.05). Uppercase letters: comparison among days within each treatment; Lowercase letters: comparison among treatments within each day; *Italic letters*: significant difference among treatments within each day.
